# Salmonella Septic Arthritis in Immunocompetent Young Children: A Rare Cause of Pediatric Monoarthritis

**DOI:** 10.7759/cureus.109068

**Published:** 2026-05-17

**Authors:** Kathleen Teo Khai Ling, Imma Isniza Ismail, Norazian Kamisan

**Affiliations:** 1 Orthopedics, Universiti Putra Malaysia, Serdang, MYS

**Keywords:** acute osteomyelitis, hip and ankle monoarticular septic arthritis, hip subluxation, immunocompetent children, orthopedic emergency, pediatric septic arthritis, salmonella spp

## Abstract

Septic arthritis is an orthopedic emergency requiring prompt diagnosis and treatment to prevent irreversible joint damage. *Staphylococcus aureus* is the most common causative organism in pediatric septic arthritis. In contrast, *Salmonella* species are rare etiological agents, particularly in immunocompetent children without predisposing conditions.

We report two cases of *Salmonella*-induced septic arthritis in healthy children. The first case involved an 11-month-old girl with acute right ankle septic arthritis. The second case involved a 15-month-old boy with subacute right hip septic arthritis complicated by proximal femoral osteomyelitis and subsequent hip subluxation. Both patients underwent surgical drainage and received prolonged intravenous ceftriaxone therapy, resulting in clinical and laboratory improvement.

Although uncommon, *Salmonella* septic arthritis can occur in immunocompetent young children and may lead to significant morbidity, particularly with delayed presentation. Early recognition, prompt surgical intervention, and culture-guided antibiotic therapy are essential to optimize outcomes and minimize long-term complications.

## Introduction

Septic arthritis in children is a true orthopedic emergency, with an estimated annual incidence of approximately 1-10 cases per 100,000 children and a higher prevalence in boys, affecting infants and young children due to their immature immune systems and rich metaphyseal blood supply [1,2]. Delayed diagnosis or treatment can result in irreversible cartilage destruction, growth plate damage, and lifelong functional disability [3].

Pediatric septic arthritis most commonly arises from hematogenous spread of bacteria, with *Staphylococcus aureus* being the predominant pathogen. Other commonly implicated organisms include *Kingella kingae*, *Streptococcus* species, and, less frequently, *Haemophilus influenzae* type B, particularly in children younger than four years of age [2,3]. In contrast, *Salmonella *species, typically associated with gastrointestinal infections, rarely cause osteoarticular infections in children and have classically been described in patients with hemoglobinopathies or acquired immunodeficiencies; however, cases in immunocompetent children have also been reported [4,5].

Reports of *Salmonella* septic arthritis in immunocompetent children without identifiable risk factors remain exceedingly rare. The pathogen accounts for a small proportion of pediatric septic arthritis cases, and most published accounts are isolated case reports or small series describing this uncommon presentation [6]. *Salmonella *osteoarticular infections are well-recognized in patients with sickle cell disease but rarely manifest as bone and joint infections in immunocompetent children [3]. The hip and knee are the most commonly affected joints, whereas ankle involvement, as observed in Case 1, is particularly uncommon.

## Case presentation

Case 1

An 11-month-old previously healthy girl was referred from a different center with a two-day history of refusal to bear weight and reduced movement of the right ankle. The symptoms developed spontaneously and were associated with high-grade nocturnal fever, for which oral paracetamol had been administered. Before the illness, the child was able to stand with support and cruise along furniture. There was no history of recent infection, travel, insect bite, trauma, or recent vaccination.

At the referring hospital, she received one day of intravenous ampicillin-sulbactam, which resulted in defervescence, but her ankle symptoms persisted. On examination at our center, her temperature was 37.7°C, and she was alert and irritable during examination of the right ankle. The ankle was swollen, erythematous, and held in plantarflexion, with marked pain on palpation. Movements of the knee and hip were preserved.

**Figure 1:**
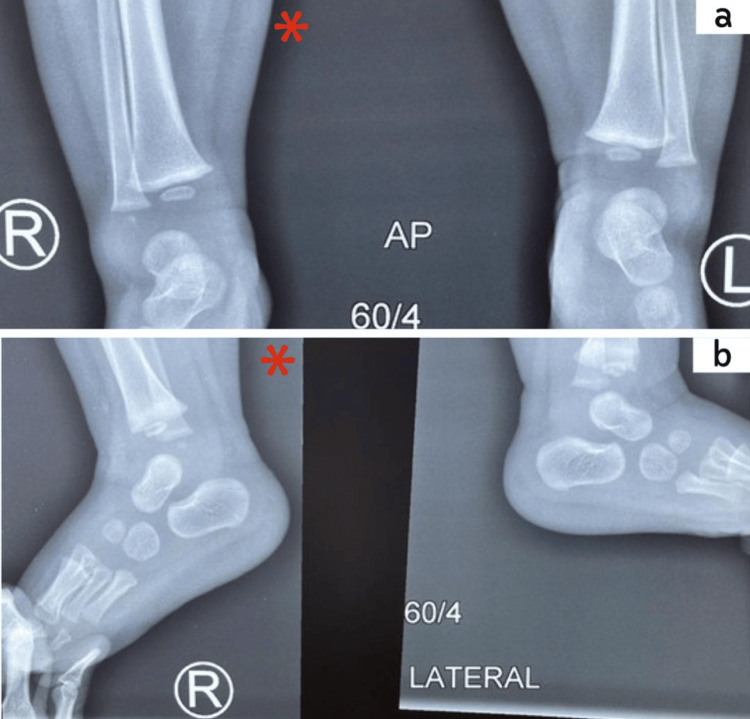
Plain radiographs of both the ankles in anteroposterior and lateral views, labeled a and b, respectively, pathological right ankle, marked with red asterisk, showing no bony abnormality or joint space widening at initial presentation

Laboratory investigations revealed a white blood cell count of 13,000/mm³ (normal value: 5,500-15,000/mm³) and a C-reactive protein (CRP) level of 24 mg/dL (normal value: <10 mg/dL). Plain radiographs of the ankle were unremarkable (Figure 1), while ultrasonography demonstrated a joint effusion with debris. Given the strong clinical suspicion of septic arthritis, urgent surgical arthrotomy was performed, draining approximately 5 mL of purulent synovial fluid. Culture of the synovial fluid grew *Salmonella* spp., while blood, urine, and stool cultures were negative.

Due to persistently raised inflammatory markers post-index washout, a second surgical washout was performed. The patient showed marked clinical improvement and began bearing weight on postoperative day five. Following the infectious disease consultation, hematogenous spread was considered the likely source of *Salmonella* septic arthritis. Evaluation for alternative infective foci, including cerebrospinal fluid analysis, abdominal ultrasonography, and echocardiography, was unremarkable. Screening for hemoglobinopathy and immunodeficiency, including HIV, was negative. The patient completed a six-week course of intravenous ceftriaxone with normalization of inflammatory markers and good clinical recovery.

Case 2

A 15-month-old boy was referred for further management of partially treated *Salmonella *bacteriemia with right hip septic arthritis, as the child refused to bear weight. The illness began during an episode of *Influenza A* infection, accompanied by transient loose stools that were treated symptomatically. Although the fever resolved with recovery from influenza, the child continued to exhibit progressive limitation of movement in the right lower limb.

Parents reported difficulty moving the child’s right leg during diaper changes due to pain and crying. On examination, the child was afebrile but irritable. He stood bearing weight on the left lower limb, with the right hip and knee held in slight flexion. The right hip was tender on palpation, with no obvious swelling.

**Figure 2:**
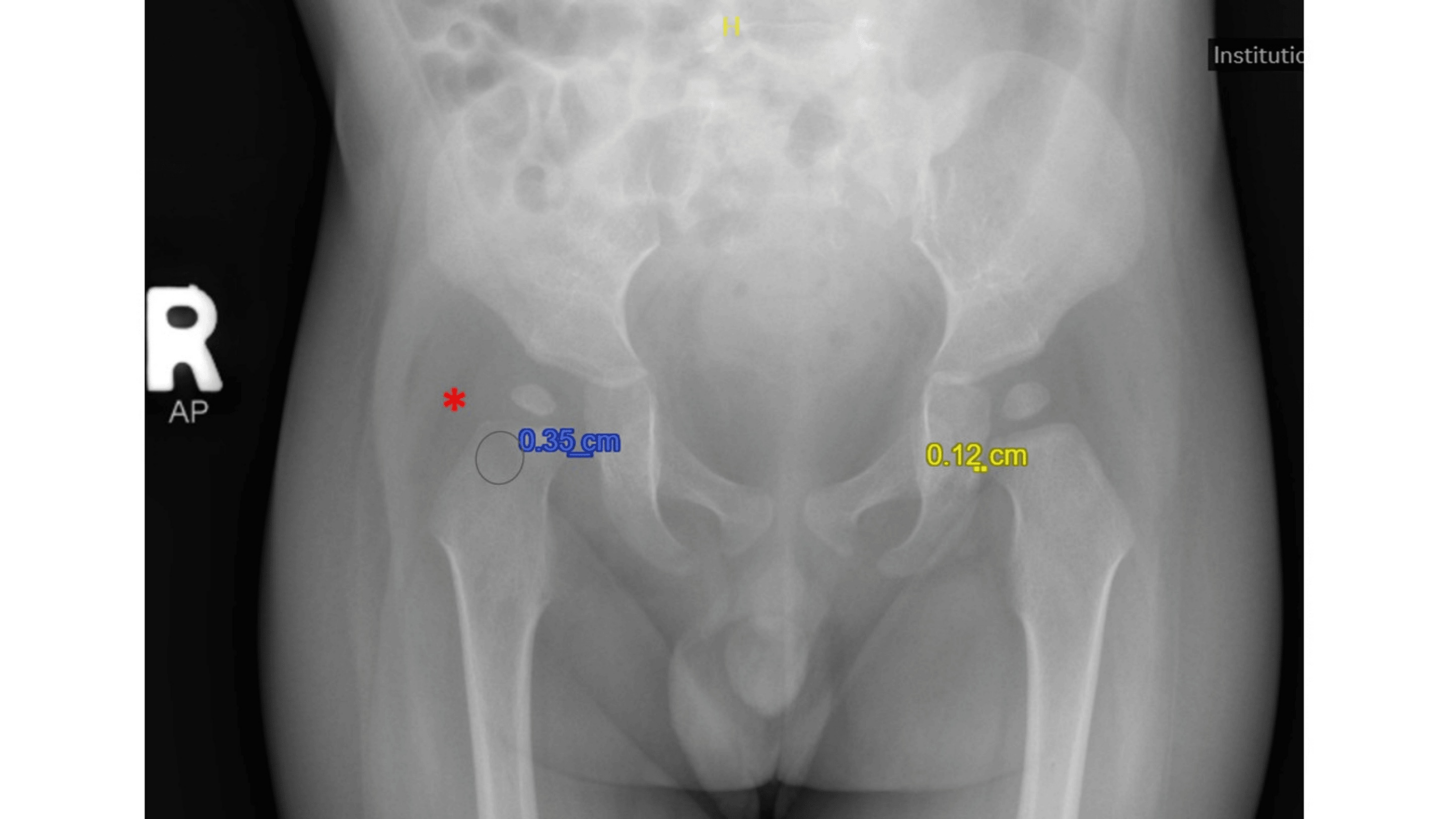
Anteroposterior radiograph of the pelvis. The image demonstrates widening of the right hip joint space, marked with red asterisk with metaphyseal rarefaction of the proximal femur (circled), suggestive of septic arthritis with associated osteomyelitis

Laboratory investigations showed a white blood cell count of 14,600/mm³ and a CRP level of 77 mg/dL. Blood culture grew *Salmonella *spp. Plain radiographs of the pelvis demonstrated right hip joint space widening with metaphyseal rarefaction of the proximal femur (Figure 2). Ultrasonography confirmed a hip effusion. A diagnosis of subacute septic arthritis of the right hip with concomitant acute osteomyelitis of the proximal femur was made.

**Figure 3:**
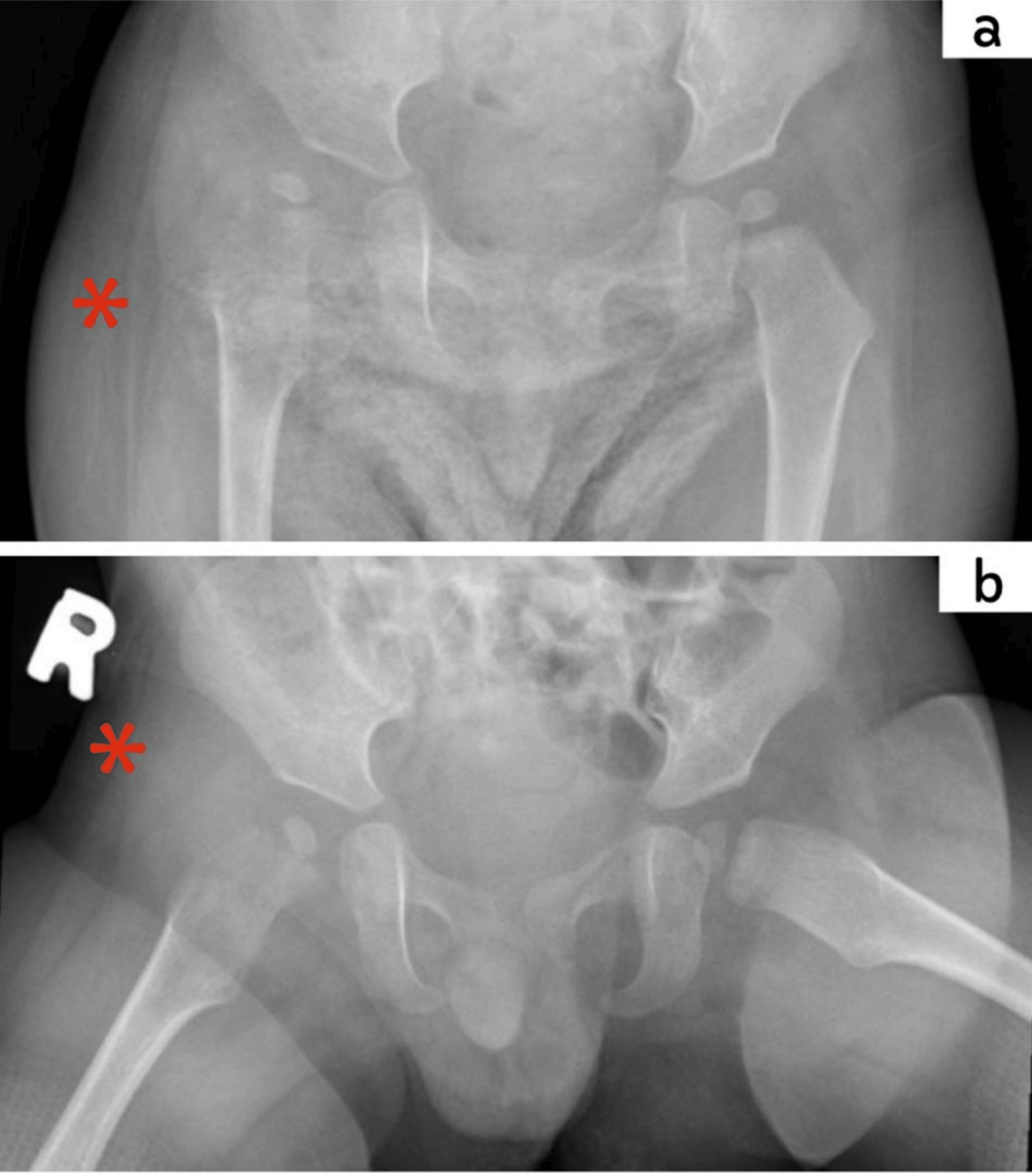
Anteroposterior radiograph of the pelvis with the right hip, marked with red asterisk following surgical arthrotomy, labeled (a) showing subluxation of the right hip joint is evident secondary to capsular and joint destruction. Subsequently, the hip has been reduced and maintained with hip spica application, labeled (b)

Urgent surgical arthrotomy yielded approximately 5 mL of turbid synovial fluid with sediments, and intraoperative findings included thinning of the hip capsule. Synovial fluid cultures were negative, as were urine and stool cultures. Histopathology of the right hip synovium showed granulation tissue. The full blood picture revealed mild anemia, likely nutritional, with no evidence of sickle cells; leukocyte changes were consistent with infection. Echocardiography was normal. Postoperatively, a plain radiograph demonstrated subluxation of the affected hip, which was managed with closed reduction and hip spica application (Figure 3). 

**Figure 4:**
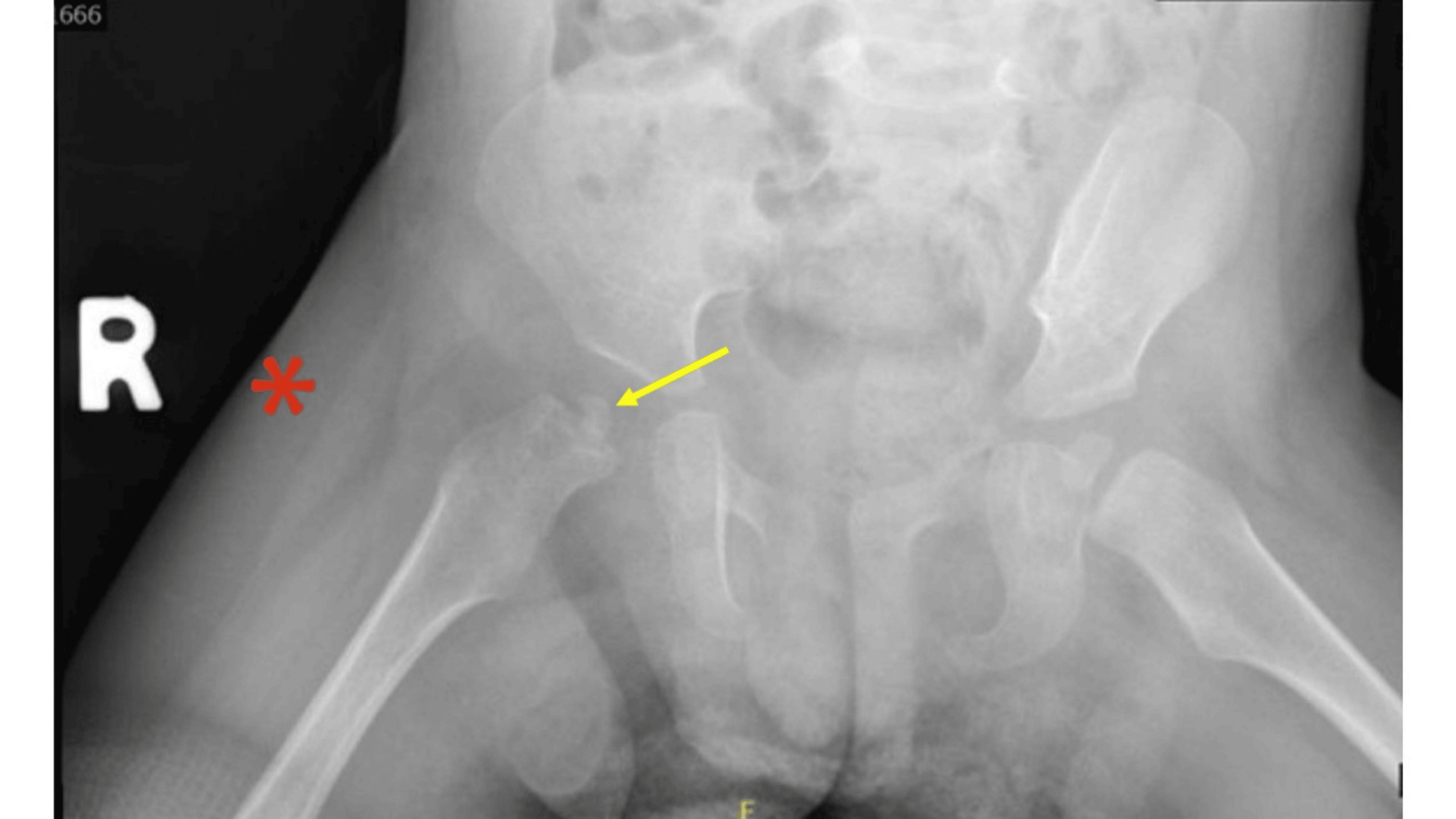
Anteroposterior radiograph of the pelvis. There is evidence of right (red asterisk) femoral head destruction, marked with yellow arrow, consistent with advanced joint damage following septic arthritis

The child received a four-week course of intravenous ceftriaxone, with improvement in symptoms and inflammatory markers. However, follow-up imaging revealed destruction of the femoral head (Figure 4), indicating significant joint damage despite appropriate treatment. The patient was planned for pediatric medical follow-up to monitor growth and development, with further evaluation for immunodeficiency or failure to thrive if clinically indicated.

## Discussion

Nontyphoidal *Salmonella *is transmitted via the fecal-oral route, typically through contaminated food or water, and commonly presents as a self-limiting gastroenteritis within 8-72 hours. However, although bacteremia occurs in fewer than 5% of healthy individuals, it can lead to severe, potentially fatal bloodstream infections, particularly in immunocompromised patients [7]. Hematogenous dissemination to the synovium or metaphyseal region can result in septic arthritis or osteomyelitis, as demonstrated in our cases.

Clinical features of pediatric septic arthritis include acute joint pain, limited range of motion, and refusal to bear weight [8]. Laboratory markers such as elevated C-reactive protein and leukocytosis support the diagnosis but are not specific [2,3]. Ultrasonography is a sensitive modality for detecting joint effusions, whereas early radiographs may appear normal [2,3]. The definitive diagnosis depends on synovial fluid culture, which remains the gold standard.

Prompt surgical drainage and appropriate antibiotic therapy are essential to prevent cartilage destruction and long-term disability. Third-generation cephalosporins, such as ceftriaxone, remain the treatment of choice for *Salmonella *infections, with recommended treatment durations of two to four weeks depending on disease severity and clinical response [9]. These cases illustrate the wide spectrum of disease severity, underscoring the importance of early recognition and intervention.

## Conclusions

*Salmonella* septic arthritis, though rare, can occur in previously healthy young children and may result in significant morbidity, particularly with delayed or subacute presentation. Clinicians should maintain a high index of suspicion for atypical pathogens in children with monoarthritis unresponsive to empirical therapy. Early surgical drainage and culture-guided antibiotic treatment are critical to optimizing outcomes.
